# Improvement of digital PCR conditions for direct detection of KRAS mutations

**DOI:** 10.1002/jcla.23344

**Published:** 2020-04-24

**Authors:** Jina Lee, Ji Hyun Kim, Sun Hyung Kang, Hee Min Yoo

**Affiliations:** ^1^ Center for Bioanalysis Korea Research Institute of Standards and Science (KRISS) Daejeon Korea; ^2^ College of Pharmacy Chungnam National University Daejeon Korea; ^3^ Division of Gastroenterology and Hepatology Department of Internal Medicine Chungnam National University School of Medicine Daejeon Korea

**Keywords:** colorectal cancer, Digital PCR (dPCR), genomic DNA (gDNA), KRAS, nucleosomal DNA (nDNA)

## Abstract

**Background:**

In standard analytical conditions, an isolation step is essential for circulating tumor DNA (ctDNA) analysis. The necessity of this step becomes unclear with the development of highly sensitive detection methods. The aim of this study was to evaluate ctDNA mimetic nDNA detection as reference materials (RMs) using dPCR technologies either directly from serum or without serum.

**Methods:**

To determine an absolute count of both mutation and wild‐type bearing DNA molecules, genomic DNA (gDNA) and nucleosomal DNA (nDNA), which are similar in size to cell‐free DNA, were evaluated. We tested 3 KRAS mutations in colorectal cancer cell lines.

**Results:**

We describe the recent progress in RMs. The short DNA fragments, such as sDNA and nDNA, exhibited higher quantitative values of dPCR compared to gDNA. The efficiency between Atlantis dsDNase (AD) and Micrococcal Nuclease (MN) affects DNA quantification. Moreover, there was a significant difference in dPCR output when spiking gDNA or nDNA containing KRAS mutations into FBS compared to the dPCR output under non‐FBS conditions.

**Conclusion:**

The matrix effect crucially affects the accuracy of gDNA and nDNA level estimation in the direct detection of mimic of patient samples. The form of reference material we proposed should be optimized for various conditions to develop reference materials that can accurately measure copy number and verify the detection of KRAS mutations in the matrix.

## 1 INTRODUCTION

Colorectal cancer (CRC) is the third most common form of cancer in both men and women, with over one million cases diagnosed each year worldwide.^1^ Approximately, 30%‐50% of colorectal tumors are known to have a mutated *KRAS* gene, predominantly found in codons 12 and 13, indicating that up to 50% of patients with colorectal cancer may respond to anti‐epidermal growth factor receptor (EGFR) antibody therapy such as cetuximab.^2^ In the era of targeted therapy for cancer, KRAS testing is utilized in the initial diagnosis of colorectal cancer.

Liquid biopsy is non‐invasive means of molecular diagnostics in the clinical field.^3^, ^4^, ^5^, ^6^ The detection and analysis of circulating cell‐free DNA (cfDNA) in the blood has emerged as an alternative analytic method with the potential to provide efficient characterizations of cancer genomes in real time.^7^, ^8^ Previous observations of cfDNA fragment size distributions had peaks corresponding to DNA associated with nucleosomes (~150 bp). DNA is protected from nuclease digestion through its association with a nucleosome core particle (NCP). Moreover, nucleosome occupancy could be used as a footprint to determine the tissue of origin of cfDNA.^9^, ^10^ Analysis of ctDNA from the plasma or serum of cancer patients has been widely used to detect cancer‐related single nucleotide variants (SNV) and copy number alterations (CNA) for the purpose of monitoring treatment response to chemotherapy.^11^, ^12^

For the past several decades, quantitative polymerase chain reactions (qPCR) have become the gold standard for quantifying gene expressions. The recently developed digital polymerase chain reaction (dPCR) enables the absolute quantitation of nucleic acids in a sample.^13^, ^14^ dPCR does not require calibration with qualified standards for comparison. However, DNA quantity should be metrologically traceable to a reference.^15^, ^16^ To date, many nucleic acid quantitation methods have been developed, such as enumeration‐based flow cytometric (FCM) counting. Chemical analysis methods based on isotope‐dilution mass spectrometry (IDMS) and capillary electrophoresis (CE) can be accurately calibrated with solutions of DNA.^17^, ^18^ Moreover, an international comparison study was conducted between metrology institutes using the dPCR method.^19^ More recently, the droplet digital PCR (ddPCR) method was developed as a powerful analytical technique for clinical applications.^20^, ^21^ For example, ddPCR can be used to detect somatic mutations, amplifications, and deletions of specific genes.^22^, ^23^ DNA size and concentration are considerable factors that affect the reliability of measurement results. The influence of the matrix effect on dPCR was observed due to the high levels of sensitivity. However, there are only a few research reports that have directly tested the matrix effect. In one pilot study, which aimed to evaluate ctDNA detection using the dPCR platform, ctDNA was detected in metastatic colorectal cancer (mCRC) patients directly from plasma as well as after an isolation step.^24^ In this study, we conclude that optimized conditions are required to increase the precision of ddPCR to develop reference materials with matrix conditions.

## 2 MATERIALS AND METHODS

### 2.1 Cell lines

Cell lines RKO (KRAS WT), Ls174T (KRAS G12D), SW480 (KRAS G12V), and HCT‐116 (KRAS G13D) were obtained from the American Type Culture Collection (ATCC). The culture medium for each cell line was determined according to the information provided by ATCC. The cell lines were cultured in a humidified atmosphere of 5% CO_2_ at 37°C. The subcultures were produced with a ratio of 1:5 when the cell density reached 80%‐90% every 3 or 4 days.

### 2.2 DNA extraction

Genomic DNA (gDNA) was extracted from each cell line using the DNeasy Blood & Tissue kit (QIAGEN) according to the manufacturer's instructions. The purity of the extracted gDNA was checked by measuring the absorbance at 260 nm (A260), 280 nm (A280), and 230 nm (A230) with a Nanodrop 2000 spectrophotometer. Extracted gDNA with a A260/A280 ratio between 1.8 and 2.0 and a A260/A230 ratio over 2.0 were considered satisfactory to produce template DNA for dPCR. Nucleosomal DNA from cell lines was captured and purified with the EZ Nucleosomal DNA Prep kit (Zymo Research) according to the manufacturer's protocol. We spiked gDNA or nDNA into fetal bovine serum (FBS) and used it directly for the PCR reaction without purification to exclude purification efficiency.

### 2.3 Droplet digital PCR measurement

A duplex ddPCR analysis was performed for all experiments using a QX200 system (BioRad Laboratories, Inc). The reaction mixture had a volume of 20 µL and comprised of 10 µL of 2× ddPCR Super Mix for Probe, 4 µL of 0.25 µM primer mixture, 1 µL of 0.25 µM wild‐type probe labeled with HEX (GenoTech), 1 µL of 0.25 µM mutant probe labeled with FAM (GenoTech), 2 µL of ddH2O, and 2 µL of template DNA with a concentration of 25 ng/µL. The PCR cycling conditions were 95℃ for 10 min, followed by 40 cycles of 94℃ for 30 s and 58℃ for 60 s (54℃ for KRAS G12V), with a final 10‐min incubation stage at 98℃.

### 2.4 Oligonucleotides

Oligonucleotide primers and probes were synthesized by GenoTech to HPLC‐grade. Primers were designed using the Primer3 software. The primers used for each gene are provided in Table 1. Probes were labeled with either FAM (F) or HEX (H).

**TABLE 1:** Primers and fluorogenic probes used in this study

Cell type	KRAS genotype	Primer sequence	Probe sequence
RKO	WT	Forward 5′ ‐ AGGCCTGCTGAAAATGACTGAATAT ‐ ′3 Reverse 5′ ‐ GCTGTATCGTCAAGGCACTCTT‐ 3′	TTGGAGCTGGTGGCGT (with G12D, G13D) TAGTTGGAGCTGGTGGCGTAGGC (with G12V)
Ls174T	G12D (c.35G>A)		TGGAGCTGATGGCGT
SW480	G12V (c.35G>T)		TGGAGCTGTTGGCGT
HCT‐116	G13D (c.38G>A)		CTGGTGACGTAGGCA

### 2.5 Data analysis

Droplet fluorescence data were initially analyzed using the QuantaSoft software (BioRad Laboratories, Inc, version 1.7). Raw data (ie, the fluorescence values for the droplets) were exported from the QuantaSoft software to Microsoft Excel 2016. Depending on the separation of positive and negative droplets, an objective separation value k was calculated automatically.

## 3 RESULTS

In this study, we evaluated the quantification of various sizes of DNA molecules using dPCR. We also analyzed copy number value with various types of nucleases. Although estimated copies based on an absolute count of molecules is the defined measurement unit used for this methodology, we additionally compared the copy numbers of different‐sized DNA with or without matrices. Information on the primer and probe sequence is shown in Table 1.

### 3.1 Development and validation of digital PCR assays for measuring DNA fragmentation

**FIGURE 1:**
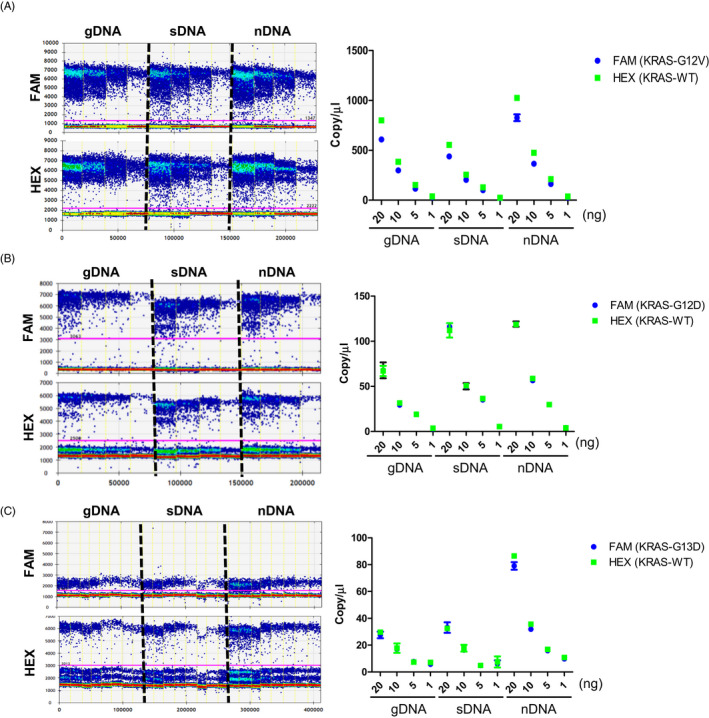
Comparison of the copy number of KRAS mutations for various sizes of DNA obtained using digital PCR. Three different sizes of genomic DNA (gDNA), sonicated DNA (sDNA), and nucleosomal DNA (nDNA) were used with various concentrations of the template to measure KRAS mutations. (A) Copy number of KRAS WT and G12V in SW480. (B) Copy number of KRAS WT and G12D in Ls174T. (C) Copy number of KRAS WT and G13D in HCT‐116

Genomic DNA and fragmented DNA can be measured with dPCR. To measure the copy number concentration of the KRAS mutations, we designed a set of dPCR assays to quantify targets of different sizes within an overlapping genomic region. As a result of performing dPCR under various concentration conditions (20ng, 10ng, 5ng, 1ng) of the template, it was confirmed that the copy number dramatically changed when sonicated DNA (sDNA) was used compared to when the gDNA and nDNA of SW480 cells were used (Figure 1A). As a result of quantifying the G12D mutation of Ls174T cells, sDNA and nDNA copy numbers tended to be higher than the gDNA value (Figure 1B), and the copy number of nDNA was confirmed to be significantly higher in the G13D mutation of HCT‐116 cells (Figure 1C). In summary, it was confirmed that short DNA fragments, such as sDNA and nDNA, exhibited higher quantitative values of dPCR compared to gDNA.

### 3.2 The efficiency of nuclease on DNA quantification

**FIGURE 2:**
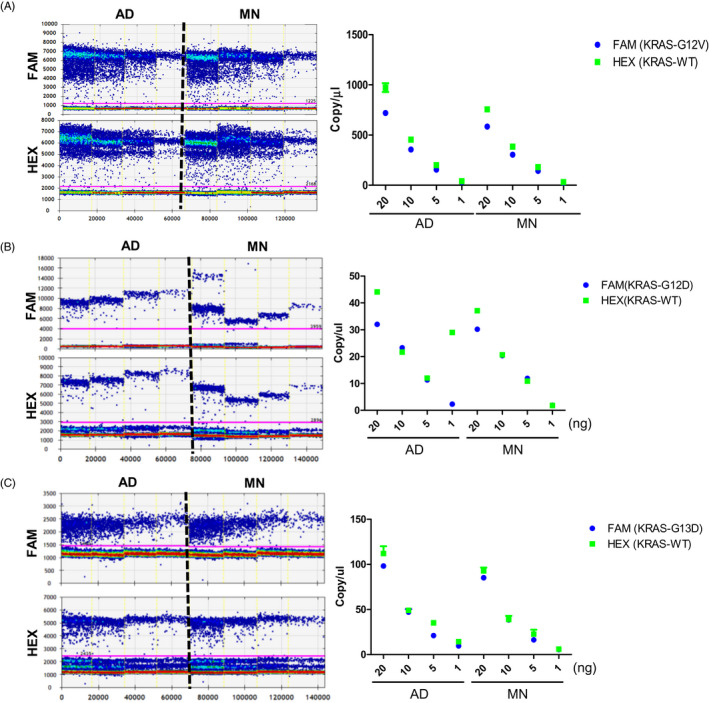
Effect of nuclease efficiency on digital PCR quantification of nucleosomal DNA. The left part is the 1D amplitude of dPCR. Blue represents KRAS mutation and green represents KRAS WT. The copy number is shown depending on the type of nuclease using gDNA in SW480 (G12V) (A), Ls174T (G12D) (B), and HCT‐116 (G13D) (C)

We used serial dilutions of gDNA to determine whether the efficiency between Atlantis dsDNase (AD) and Micrococcal Nuclease (MN) affects DNA quantification. It was confirmed that the copy numbers of the KRAS G12V, G12D, and G13D mutations significantly changed in SW480, Ls174T, and HCT‐116 cells depending on the type of nuclease. When the same concentration of DNA was used, there was a significant difference in the number of copies between AD and MN. It was also confirmed that the quantitative value was different due to the difference in the region cut by the nucleosome unit (Figure 2A‐C).

### 3.3 Matrix effect of gDNA from tumor cell lines for detecting mutations

**FIGURE 3:**
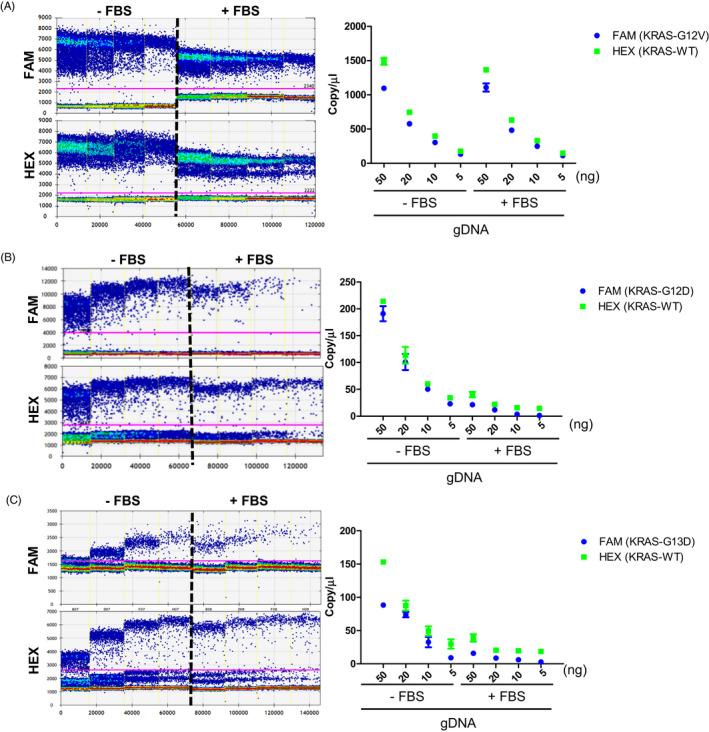
Matrix effect on dPCR quantification with gDNA as a template. The copy number is shown depending on concentration with or without a matrix using gDNA in SW480 (G12V) (A), Ls174T (G12D) (B), and HCT‐116 (G13D) (C)

The matrix effect was evaluated using gDNA spiked into fetal bovine serum (FBS). Our findings showed that the matrix effect was observed in the KRAS mutations. For example, there was a significant difference in dPCR output when spiking 50 ng of gDNA containing G12V into FBS compared to the dPCR output under non‐FBS conditions (Figure 3A). When we tested the G12D mutation, the difference in ddPCR output was greater than 10‐fold when spiking 50ng of gDNA into FBS compared to the non‐FBS conditions (Figure 3B). A similar effect was observed when we tested the detection of the G13D mutation by spiking gDNA into FBS (Figure 3C).

### 3.4 Matrix effect of nDNA from tumor cell lines for detecting mutations

**FIGURE 4:**
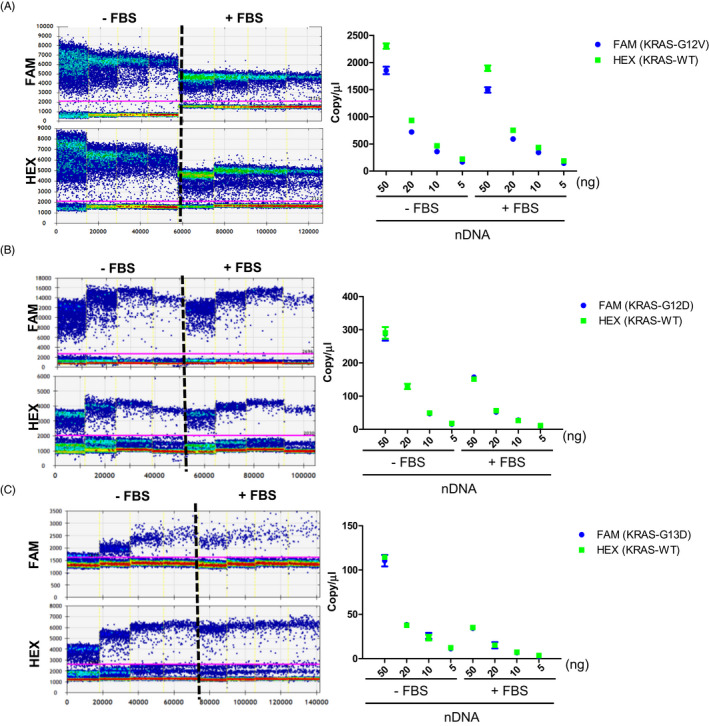
Matrix effect on dPCR quantification with nDNA as a template. The copy number is shown depending on concentration with or without a matrix using gDNA in SW480 (G12V) (A), Ls174T (G12D) (B), and HCT‐116 (G13D) (C)

To evaluate the matrix effect of nDNA spiked into fetal bovine serum (FBS), a similar experiment was performed. When we tested the KRAS mutations G12D, G12V, and G13D, there was a two to three‐fold difference in dPCR copy concentrations when using FBS compared to the non‐FBS conditions. The difference in dPCR output for G12D and G12V mutations was greater than twofold when spiking 50ng of nDNA into FBS compared to the non‐FBS conditions (Figure 4A,B). A similar effect was observed when we spiked 50 ng of nDNA containing the G13D mutation into FBS (Figure 4C).

## 4 DISCUSSION

To our knowledge, there are only a few related studies that reported nucleosomal DNA evaluation using dPCR directly from unpurified plasma.^24^ To determine the copy number difference of both mutation and wild‐type bearing DNAs, evaluations were conducted for genomic DNA (gDNA) and nDNA, which are similar in size to cell‐free DNA. In summary, the size of the DNA is a critical factor in measuring absolute copy number according to our tests with various concentrations. There were differences in the copy number of nDNA when we used two different nucleases from the kit. Copy number significantly changed when using two types of nucleases, so it is necessary to check the nuclease when measuring absolute quantifying nDNA used for reference materials. Next, we compared the copy number between gDNA and nDNA, which provided variable values for the detection of KRAS mutations with or without the serum matrix. Based on the results, DNA size, the type of enzymes, and the matrix effect should be considered when evaluating the copy number of reference materials with dPCR. For future development, we are seeking to improve the temperature distribution when designing assays with a highly parallel evaluation of primer candidates across other cancer mutations.^25^ The aforementioned verification steps would improve measurements of the copy number of customized reference materials with various mutations.^26^

## 5 CONCLUSION

In conclusion, our results show that the number of copies by dPCR between genomic DNA and fragmented DNA drived from colorectal cancer cells varies dramatically according to the location of the nuclease cleavage site. In addition, our results demonstrate that the direct detection of gDNA and nDNA of KRAS mutations mixed with serum had qualitative differences compared to the unmixed control. Moreover, with regard to the variation in copy number between the samples, our results suggest that the matrix effect critically affects the accuracy of gDNA and nDNA level estimation in the direct detection of mimic of patient samples. The form of reference material we proposed should be optimized for various conditions to develop reference materials that can accurately measure copy number and verify the detection of KRAS mutations in matrices.

## CONFLICT OF INTEREST

The authors declare that they have no competing interests.

## AUTHOR CONTRIBUTIONS

J. L., J. H. K., and H. M. Y. conceived and designed the experiments; J. L., J. H. K., performed experiments and H. M. Y. wrote this manuscript; J. L., and H. M. Y. analyzed the data; J. L., S. H. K., and H. M. Y. revised this paper.

## References

[1] Siegel RL , Miller KD , Jemal A . Cancer statistics, 2018. CA Cancer J Clin. 2018;68:7‐30. 29313949 10.3322/caac.21442

[2] Dong L , Wang S , Fu B , Wang J . Evaluation of droplet digital PCR and next generation sequencing for characterizing DNA reference material for KRAS mutation detection. Sci Rep. 2018;8(1):1‐9. 30504843 10.1038/s41598-018-27368-3 PMC6269532

[3] Brychta N , Krahn T , Von Ahsen O . Detection of KRAS mutations in circulating tumor DNA by digital PCR in early stages of pancreatic cancer. Clin Chem. 2016;62:1482‐1491. 27591291 10.1373/clinchem.2016.257469

[4] Orue A , Rieber M . Optimized multiplex detection of 7 KRAS Mutations by Taqman Allele‐SpecificqPCR. PLoS ONE. 2016;11:1‐13. 10.1371/journal.pone.0163070 PMC5025196 27632281

[5] Denis JA , Patroni A , Guillerm E et al Droplet digital PCR of circulating tumor cells from colorectal cancer patients can predict KRAS mutations before surgery. Mol Oncol. 2016;10:1221‐1231. 27311775 10.1016/j.molonc.2016.05.009 PMC5423194

[6] Lopez SO , Garcia‐Olmo DC , Garcia‐Arranz M , Guadalajara H , Pastor C , Garcia‐Olmo D . KRAS G12V mutation detection by droplet digital PCR in circulating cell‐free DNA of colorectal cancer patients. Int J Mol Sci. 2016;17:1‐9. 10.3390/ijms17040484 PMC4848940 27043547

[7] Wan JCM , Massie C , Garcia‐Corbacho J et al Liquid biopsies come of age: towards implementation of circulating tumour DNA. Nat Rev Cancer. 2017;17:223‐238. 28233803 10.1038/nrc.2017.7

[8] Sclafani F , Chau I , Cunningham D et al KRAS and BRAF mutations in circulating tumour DNA from locally advanced rectal cancer. Sci Rep. 2018;8(1):1‐9. 29362371 10.1038/s41598-018-19212-5 PMC5780472

[9] Snyder MW , Kircher M , Hill AJ , Daza RM , Shendure J . Cell‐free DNA comprises an in vivo nucleosome footprint that informs its tissues‐of‐origin. Cell. 2016;164:57‐68. 26771485 10.1016/j.cell.2015.11.050 PMC4715266

[10] Lai WKM , Pugh BF . Understanding nucleosome dynamics and their links to gene expression and DNA replication. Nat Rev Mol Cell Biol. 2017;18(9):548‐562. 28537572 10.1038/nrm.2017.47 PMC5831138

[11] Nakagama H , Hayashi H , Okusaka T et al Clinical utility of circulating tumor DNA for molecular assessment in pancreatic cancer. Sci Rep. 2015;5:1‐10. 10.1038/srep18425 PMC4680882 26669280

[12] Siravegna G , Marsoni S , Siena S , Bardelli A . Integrating liquid biopsies into the management of cancer. Nat Rev Clin Oncol. 2017;14:1‐18. 28252003 10.1038/nrclinonc.2017.14

[13] Hindson BJ , Ness KD , Masquelier DA , et al High‐throughput droplet digital PCR system for absolute quantitation of DNA copy number. Anal Chem. 2011;83(22):8604‐8610. 22035192 10.1021/ac202028g PMC3216358

[14] Hindson CM , Chevillet JR , Briggs HA et al Absolute quantification by droplet digital PCR versus analog real‐time PCR. Nat Methods. 2013;10:1003‐1005. 23995387 10.1038/nmeth.2633 PMC4118677

[15] Whale AS , Jones GM , Pavšič J et al Assessment of digital PCR as a primary reference measurement procedure to support advances in precision medicine. Clin Chem. 2018;64(9):1296‐1307. 29903874 10.1373/clinchem.2017.285478

[16] Bhat S , Emslie KR . Digital polymerase chain reaction for characterisation of DNA reference materials. Biomol Detect Quantif. 2016;10:47‐49. 27990349 10.1016/j.bdq.2016.04.001 PMC5154631

[17] Kwon HJ , Jeong JS , Bae YK , Choi K , Yang I . Stable isotope labeled DNA: A new strategy for the quantification of total DNA using liquid chromatography‐mass spectrometry. Anal Chem. 2019;91(6):3936‐3943. 30773003 10.1021/acs.analchem.8b04940

[18] Yoo HB , Park SR , Dong L et al International comparison of enumeration‐based quantification of DNA copy‐concentration using flow cytometric counting and digital polymerase chain reaction. Anal Chem. 2016;88:12169‐12176. 28193036 10.1021/acs.analchem.6b03076

[19] Corbisier P , Vincent S , Schimmel H et al CCQM‐K86/P113.1: relative quantification of genomic DNA fragments extracted from a biological tissue. Metrologia. 2012;49(1A):08002.

[20] Miotke L , Lau BT , Rumma RT , Ji HP . High sensitivity detection and quantitation of DNA copy number and single nucleotide variants with single color droplet digital PCR. Anal Chem. 2014;86:2618‐2624. 24483992 10.1021/ac403843j PMC3982983

[21] Pinheiro LB , Coleman VA , Hindson CM et al Evaluation of a droplet digital polymerase chain reaction format for DNA copy number quantification. Anal Chem. 2012;84:1003‐1011. 22122760 10.1021/ac202578x PMC3260738

[22] Decraene C , Silveira AB , Bidard FC et al Multiple hotspot mutations scanning by single droplet digital PCR. Clin Chem. 2018;64:317‐328. 29122835 10.1373/clinchem.2017.272518

[23] Milosevic D , Mills JR , Campion MB et al Applying standard clinical chemistry assay validation to droplet digital PCR quantitative liquid biopsy testing. Clin Chem. 2018;64(12):1732‐1742. 30237149 10.1373/clinchem.2018.291278

[24] Sefrioui D , Beaussire L , Perdrix A et al Direct circulating tumor DNA detection from unpurified plasma using a digital PCR platform. Clin Biochem. 2017;50:963‐966. 28645720 10.1016/j.clinbiochem.2017.06.005

[25] Cayuela JM , Mauté C , Fabre AL et al A novel method for room temperature distribution and conservation of RNA and DNA reference materials for guaranteeing performance of molecular diagnostics in onco‐hematology: a GBMHM study. Clin Biochem. 2015;48:982‐987. 25872147 10.1016/j.clinbiochem.2015.04.004

[26] Jing R , Wang H , Ju S , Cui M . Reference materials for molecular diagnostics: Current achievements and future strategies. Clin Biochem. 2018;56:11‐17. 29679554 10.1016/j.clinbiochem.2018.04.015

